# Sensorimotor performance after high-definition transcranial direct current stimulation over the primary somatosensory or motor cortices in men versus women

**DOI:** 10.1038/s41598-022-15226-2

**Published:** 2022-07-01

**Authors:** Yochai Swissa, Shlomi Hacohen, Jason Friedman, Silvi Frenkel-Toledo

**Affiliations:** 1 Department of Physical Therapy, Faculty of Health Sciences, Ariel University, Ariel, Israel; 2 Department of Mechanical Engineering, Ariel University, Ariel, Israel; 3 Department of Physical Therapy, Stanley Steyer School of Health Professions, Sackler Faculty of Medicine, Tel Aviv University, Tel Aviv, Israel; 4 Sagol School of Neuroscience, Tel Aviv University, Tel Aviv, Israel; 5 Department of Neurological Rehabilitation, Loewenstein Rehabilitation Medical Center, Ra’anana, Israel

**Keywords:** Learning and memory, Sensorimotor processing

## Abstract

The primary somatosensory (S1) cortex is a central structure in motor performance. However, transcranial direct current stimulation (tDCS) research aimed at improving motor performance usually targets the primary motor cortex (M1). Recently, sex was found to mediate tDCS response. Thus, we investigated whether tDCS with an anodal electrode placed over S1 improves motor performance and sensation perception in men versus women. Forty-five participants randomly received 15-min high-definition tDCS (HD-tDCS) at 1 mA to S1, M1, or sham stimulation. Reaching performance was tested before and immediately following stimulation. Two-point orientation discrimination (TPOD) of fingers and proprioception of a reaching movement were also tested. Although motor performance did not differ between groups, reaching reaction time improved in the M1 group men. Reaching movement time and endpoint error improved in women and men, respectively. Correct trials percentage for TPOD task was higher in the S1 compared to the M1 group in the posttest and improved only in the S1 group. Reaching movement time for the proprioception task improved, overall, and endpoint error did not change. Despite the reciprocal connections between S1 and M1, effects of active tDCS over S1 and M1 may specifically influence sensation perception and motor performance, respectively. Also, sex may mediate effects of HD-tDCS on motor performance.

## Introduction

Sensory inputs are necessary for the successful execution and acquisition of skillful voluntary movements^1–7^. The primary somatosensory (S1) cortex is a central structure involved in motor learning and motor performance^1,8–12^. S1 has strong reciprocal connections with the primary motor cortex (M1)^1^ and one-third of the cortico-spinal tract fibers that control movement originate from S1^13^. Physiological evidence from animal models indicates that a lesion to S1 in monkeys impaired learning of new motor behaviors^3^. In humans, the extent of contralesional M1 and S1 activity correlated with the severity of post-stroke motor deficit^14^. Disrupting somatosensation by applying inhibitory 1 Hz repetitive transcranial magnetic stimulation (rTMS) over S1 in healthy individuals prior to skilled motor practice decreased motor skill acquisition^12^.

Motor learning can be enhanced via the use of non-invasive brain stimulation methods over M1 that modulate neuroplasticity^15,16^. One non-invasive method that may improve motor performance in healthy individuals and those with neurological disorders is transcranial direct current stimulation (tDCS)^16–19^. This is a safe, easy to administer and painless stimulation method that delivers weak direct currents (usually 0.5–2 mA) through surface electrodes placed on the skull. It alters spontaneous brain activity and excitability by the subthreshold modulation of neuronal membranes in a polarity dependent manner^20^. It is commonly assumed that anodal stimulation (anodal electrode is placed over the region of interest) increases cortical excitability (inducing greater Motor Evoked Potentials (MEP), a commonly used measure of cortical excitability), while cathodal stimulation (cathodal electrode is placed over the region of interest) decreases it^15,20^; however, this is an overly simplistic assumption, and the effects of tDCS are likely to be much more complex. A non-linear dose–response relationship was found in neurophysiological^15,21–24^ and behavioral measures^25,26^. In order to avoid the assumption that anodal stimulation necessarily reflects excitatory stimulation, instead of the term anodal tDCS, we have described it in this paper as active tDCS with an anodal electrode placed over the region of interest.

In the studies which found active tDCS to be an effective means of improving motor performance in healthy participants, as well as patients suffering from neurological diseases such as stroke and Parkinson’s disease^16–19^, the anodal electrode was usually placed over M1. Considering the importance of S1 in motor learning and motor performance, stimulating S1 via tDCS may also be relevant in neurorehabilitation, especially in individuals with stroke who have a large amount of damage in M1. As recovery after stroke is attributed to plastic reorganization in the central nervous system^27,28^, enhancing the recruitment of relevant regions of interest (among them S1) for motor performance may have important clinical significance. Yet, to the best of our knowledge, only one study—that of Faraji et al.^29^—investigated the effects of tDCS over S1 on motor performance, and it was conducted in rats. They found that both unilateral and bilateral anodal somatosensory stimulation significantly improved reaching performance. It should be noted that the few tDCS studies in which healthy participants received S1 stimulation focused on sensation measures^30–34^, and indeed, some of them found improved sensation perception^30,31,33^. Also, in animals who received S1 stimulation, measures other than motor behavioral measures were mainly reported^35–37^, e.g., the application of tDCS with anode and cathode electrodes over S1 induced polarity-specific bidirectional changes in the N1 component of the sensory-evoked potentials and associated gamma oscillations^35^.

As far as we are aware, the present study is the first attempt to determine the effects of tDCS over S1 on motor performance in adults (it should be noted that some TMS studies already showed that stimulating S1 enhanced motor learning in healthy participants and individuals with chronic stroke^8,11,38^). Due to the relevance of S1 to sensation perception, we also assessed tactile sensation and proprioception as secondary outcome measures. The motor task consisted of sequential point-to-point reaching movements on a graphics tablet, a version of a similar, previously used task^25,39,40^. The sensation tasks consisted of (1) two-point orientation discrimination (TPOD) of fingers, in which the participants were required to indicate the orientation of the prongs^41^ and (2) proprioception of reaching movements, in which the participants were asked to actively reproduce reaching movements without vision after being passively guided by the examiner (a digitized version of the previously used paper and pencil task—the Brief Kinesthesia Test^42^). As stimulation of S1 via conventional tDCS, which uses large pad electrodes, delivers current to diverse brain regions rather than to the targeted region of interest only, we used high definition (HD) tDCS with optimized electrode configurations for maximal focal stimulation to S1 or M1. Improved spatial focality of tDCS can be achieved using HD-tDCS^43–46^. In comparison to conventional large pad tDCS, HD-tDCS (4 × 1 ring electrode configurations) demonstrated a peak induced electric field magnitude at the sulcus and adjacent gyri directly beneath the active electrode^44^.

In addition, we took into consideration the effect of sex, and investigated a possible interaction between sex and brain stimulation because it may mediate the effects of tDCS/HD-tDCS on cortical induced electric field current^47,48^, intracortical excitability^49^ and different behaviors^50–55^ such as visually guided reaching movements^52^ and social cognition skills^50^. In some studies, tDCS significantly affected behavior of women only and in others—in men^50,51,54^. Only in women, tDCS, in which the anodal electrode was placed over the medial prefrontal cortex, led to slower reaction times (vs. sham) for the social intention attribution task^50^, and tDCS, in which the anodal electrode was placed over the orbitofrontal cortex, increased the performance of risky decision making^54^. In men, tDCS, in which the anodal electrode was placed over the dorsolateral prefrontal cortex, enhanced practice-related changes in accuracy of response execution, but in women—prevented practice-related changes^51^. Concerning motor behavior, Gorbet and Stains^52^ found that reaction time slowed in men but not in women following inhibitory continuous theta burst stimulation over the contralateral dorsal premotor cortex during a reaching task.

Proposed explanations for sex being a factor that mediates the effect of brain stimulation on cortical excitability and behavior include hormonal levels, neurotransmitter balances and cortical bone structure^56^. Fluctuations in estradiol and progesterone across the menstrual cycle in women affect cortical excitability^56–58^. It appears that estradiol facilitates cortical excitability, likely through glutamatergic mechanisms, while progesterone metabolites dampen cortical excitability, likely through gamma-aminobutyric acid (GABA)^59^. Alterations in GABA following active tDCS are related to motor learning capacity^60,61^. Effects of hormonal fluctuations on manual dexterity are controversial^62–64^. Although Maki et al.^63^ reported that manual dexterity improved in the mid-luteal phase compared to the follicular phase, Keenan et al.^62^ reported that dexterity did not change throughout the menstrual cycle. In addition, evidence regarding the amount of induced electrical current at the cortex of men versus women are inconsistent. Whereas Russell et al.^47^ found that men would receive ~ 45% more current at the cortex than women, perhaps because of the more cancellous bone composition in men compared to women, induced electric field was found to be higher in female head models than male head models across several metrics^48^.

We hypothesized that HD-tDCS over S1 would be more effective in improving (1) motor performance than sham tDCS and (2) sensation perception than HD-tDCS over M1 and sham tDCS, and that HD-tDCS over M1 would be more effective in improving (1) sensation perception than sham tDCS and (2) motor performance than HD-tDCS over S1 and sham tDCS. We expected to find sex-related differences in the effects of active HD-tDCS over S1 and M1 on motor performance. We could not hypothesize regarding the specific influence of sex on the behavioral response due to the lack of specific relevant tDCS literature in the motor domain, the mixed results regarding the interaction between sex and tDCS with respect to other behavioral domains^50–55^ and the mixed evidence of menstrual cycle phase-related motor performance differences in women^62–64^.

## Methods

### Study design

This was a single-blind, parallel, randomized, sham-controlled study. Data were collected in a brain and motor behavior laboratory based at Ariel University, Israel. Participants were randomly assigned with a 1:1 ratio, using a random number generator in WINPEPI (by researcher SFT), to one of three groups: (1) HD-tDCS over the S1 (S1 group); (2) HD-tDCS over the M1 (M1 group); and (3) sham HD-tDCS (sham group). All participants were blinded to group allocation. To ensure blinding of participants, the stimulator monitor was hidden from the participants, and the sham stimulation increased and decreased in a ramp-like fashion (see HD-tDCS section). The researcher (YS) who administered the HD-tDCS application and measured the outcomes received allocation information via coded email from another researcher (SFT). Blinding of group allocation was maintained during the data analysis [for a similar approach see^25^]. The trial was prospectively registered at the ClinicalTrials.gov registry on 06/11/2020 with the trial registration number NCT04618614. The protocol is available on the following website:

https://register.clinicaltrials.gov/prs/app/action/SelectProtocol?sid=S000AC9A&selectaction=Edit&uid=U0005AKF&ts=2&cx=-nam3zd.

### Participants

The sample size for this study was determined based on a power analysis calculation that was conducted using G*Power version 3.1.9.7. Power analysis yielded a total sample size of 42 individuals (14 individuals per group) for the detection of a significant interaction with an assumed effect size of 0.25 and a power of 80%. To account for potential data loss, we aimed for a sample size of 15 individuals per group (in total 45 participants). Forty-five participants (24 women, 21 men; aged 24 ± 2 years) participated in the study between November 2020 and January 2021. Participants were included if they were aged between 20 and 35, were right-hand dominant and were healthy according to their report. They were excluded if they took psychiatric medications, had a history of drug abuse or dependence, had any psychiatric or neurological disorder, had a history of seizures, had metal implants in their head or had musculoskeletal deficits interfering with task performance (proper reaching performance with the left arm in sitting) [for a similar approach see^25^]. Participants signed an informed consent form prior to participating in the study. All the procedures were approved by Ariel University Institutional Review Board (approval number: AU-HEA-SFT-20190326-B) and were performed in accordance with relevant guidelines and regulations. Participants were paid $20 for their participation.

### HD-tDCS

**Figure 1:**
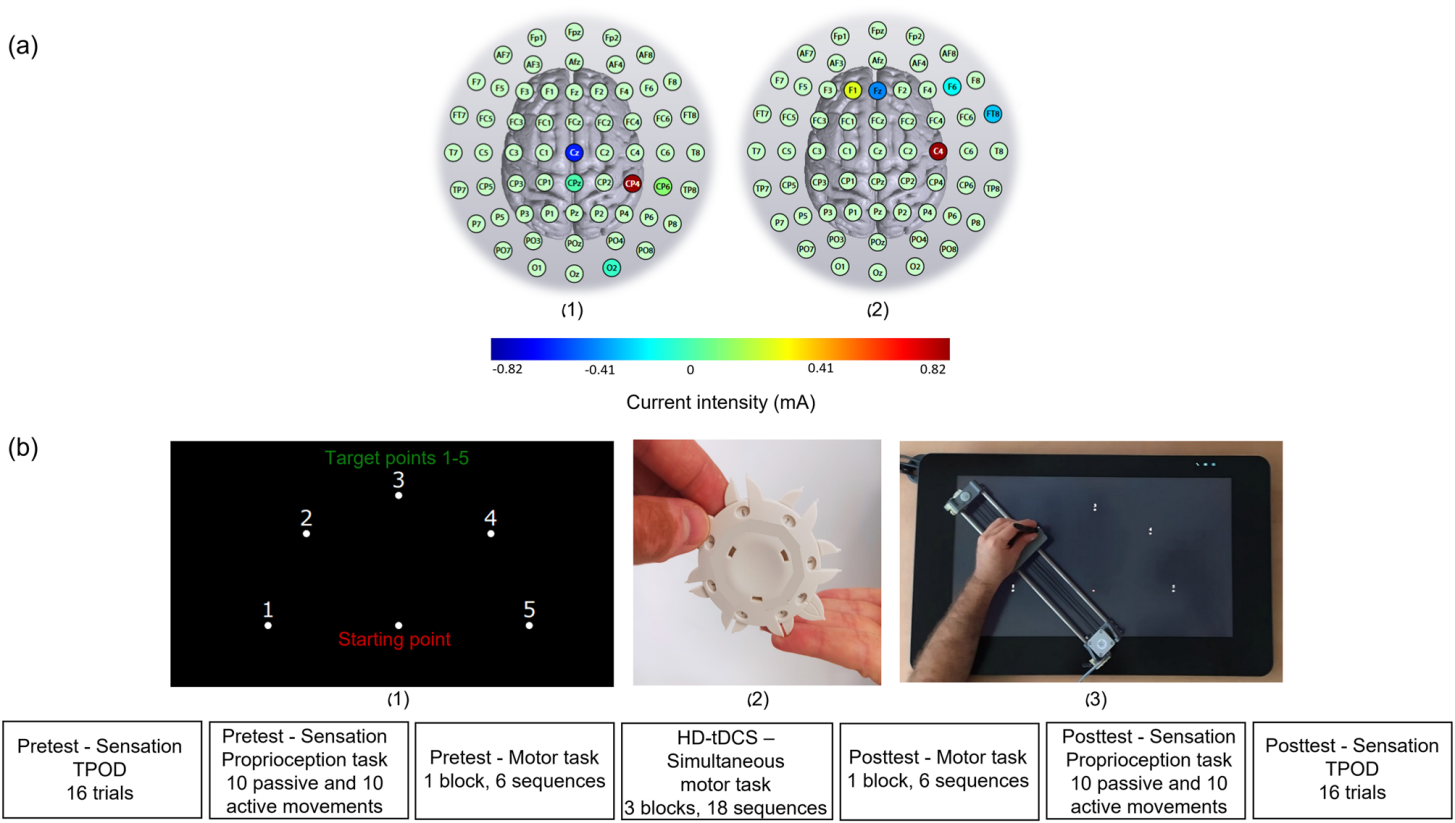
Course of study. (**a**) High-definition transcranial direct current stimulation (HD-tDCS) montage for maximal focal stimulation of the (1) right primary somatosensory cortex (S1) and (2) right primary motor cortex (M1) using the HD-Targets modelling software (Soterix Medical, New York, NY). The location and current intensity value of each stimulating electrode are shown. Red denotes anodal stimulation while blue denotes cathodal stimulation. (**b**) Experimental stimuli. (1) TPOD = Two-point orientation discrimination. The prongs were oriented randomly either across or down (trial-by-trial) to the left index and little fingers. (2) General setup of the proprioception task. The non-dominant left arm of the participant that held the stylus was placed on a custom-made device. The participants were required to reproduce a reaching movement from the starting position to target 2, with their eyes closed, after being passively moved by the device. This was repeated 10 times. (3) General setup of the motor task. Note that in this figure, for clarity, the targets and numbers are shown 3 times their relative size compared to those shown in the experiment.

**Figure 2:**
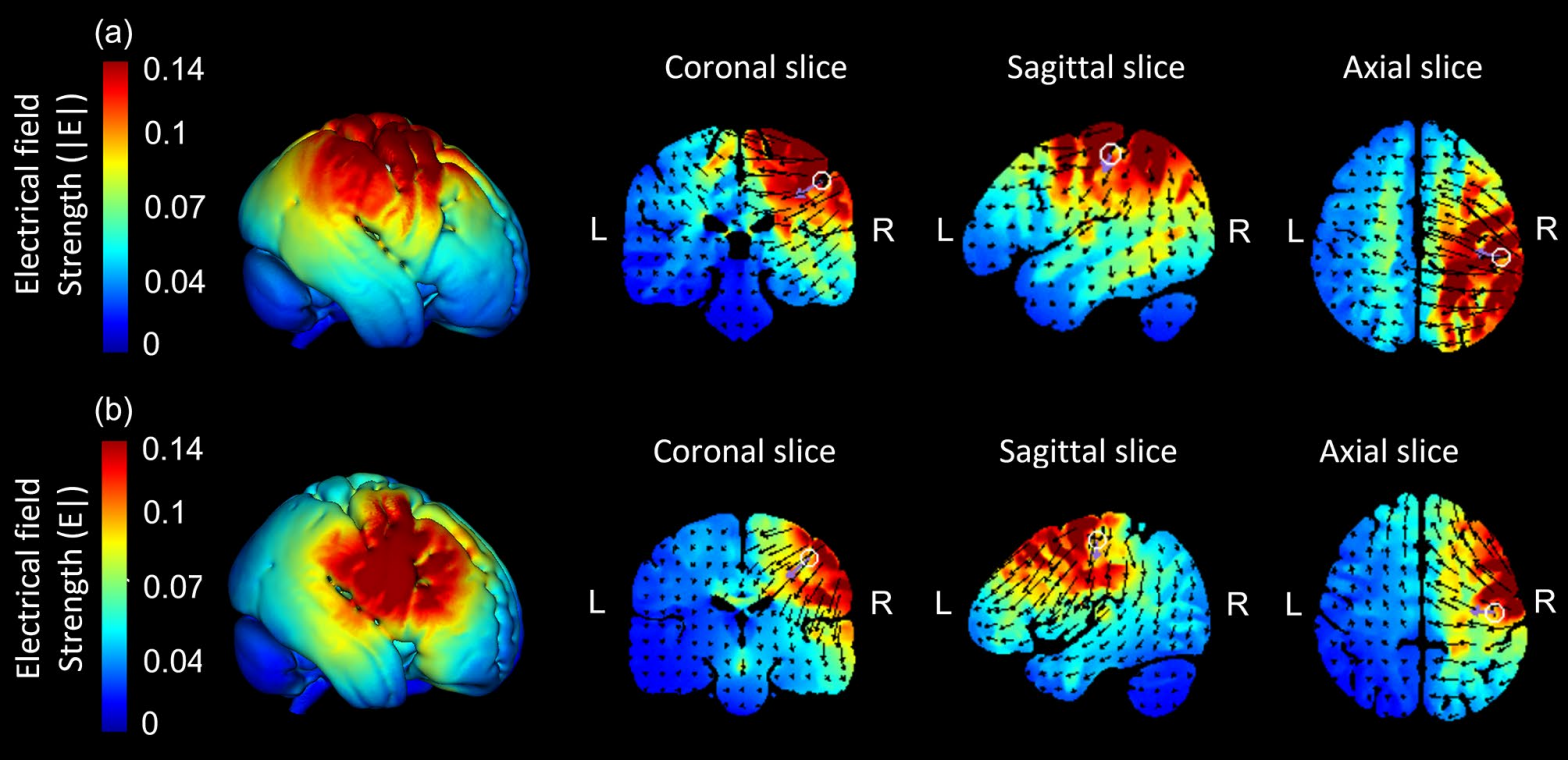
Current flow modeling during 1 mA High-definition transcranial direct current stimulation (HD-tDCS) using the HD-Target software (Soterix Medical, New York, NY). Current-flow models of (**a**) the right primary somatosensory cortex (S1) and (**b**) the right primary motor cortex (M1) are shown on 2D and 3D reconstructions of the cortical surface. Skin, skull, and cerebrospinal fluid (CSF) masks are suppressed to reveal the underlying gray matter mask. A head model derived from the MNI 152 dataset was used.

The stimulation was administered noninvasively using an M x N 9-channel high-definition transcranial electrical current stimulator from Soterix Medical (New York, NY). Five sintered Ag/AgCl electrodes were attached to plastic holders, filled with conductive gel, and embedded in a HD cap, according to the extended 10–20 method of electrode placing. We administered a single session of 15 min of active stimulation at 1 mA targeting the right S1 (primary somatosensory cortex; postcentral gyrus, based on Talairach labels) by positioning electrodes at the following sites: CP4 (0.97 mA), CP6 (0.03 mA), Cz (− 0.71 mA), CPz (− 0.12 mA), and O2 (− 0.17 mA) or right M1 (primary motor cortex, Brodmann area 4; precentral gyrus, based on Talairach labels) by positioning electrodes at the following sites: C4 (0.82 mA), Fz (− 0.43 mA), F1 (0.18 mA), F6 (− 0.24 mA), and FT8 (− 0.33 mA). HD-Targets brain modelling software (Soterix Medical, New York, NY) was used to determine the tDCS montage for maximal focal stimulation of the right S1 and right M1 (Figs. 1a, 2). In the S1 and M1 groups, the current increased in a ramp-like fashion over the course of the first 30 s, and decreased in a ramp-like fashion over the course of the last 30 s. In the sham group, the position of the electrodes was similar to the position in the S1 group. Once the current reached 1 mA over the first 30 s, it was ramped back down over 30 s. In the last min of the simulation an identical ramp up and ramp down occurred [for a similar approach see^65–67^]. Participants were asked to report any adverse effects and to rank their discomfort from 1 to 10 following one min of stimulation. If the participant felt that he does not exactly know how to define his feeling, he was asked if he means tingling or burning or itching or headache.

### Motor sequence learning task

In all participants, the non-dominant left arm was tested. The non-dominant arm was tested to challenge the motor performance of healthy participants and to allow more room for improvement in the motor performance (aiming to avoid ceiling effects). This approach is acceptable in motor learning studies [e.g.,^68,69^] and tDCS studies which investigate upper limb motor performance/learning^16,70^. After placing the tDCS cap on the head, the participants performed a sequential point-to-point movement task on the graphics tablet, a version of a similar, previously used task^39,40^. The stimuli consisted of a starting point and five targets around a semicircle (Fig. 1b1). The participants were instructed that the targets would change their colors following the sequence: 4-1-3-2-5, and to perform the task as fast and accurately as possible. A detailed description of the task is provided in a previous study^25^.

Initially, the participants were required to perform 3 sequences without errors to familiarize themselves with the task. Then, they performed the pretest which consisted of one block of 6 sequences. Two min after starting the appropriate stimulation, simultaneously with the stimulation, they performed 3 blocks of 6 sequences, i.e., 18 sequences. Brief breaks (30 s) were given between each block, while within a block breaks were not given. After finishing the tDCS stimulation, the participants performed a posttest, which was identical to the pretest (i.e., one block of 6 sequences). The stimuli were visible throughout each trial, the duration of each movement was subject-dependent (it started after the stylus was placed at the start position for 500 ms and ended when the participants reached the target and remained there for 500 ms).

Three outcome measures for the motor task were used. Movement time (s) of the reaching movements was defined as the time from movement onset (first time the tangential velocity was greater than 5% of the peak tangential velocity) until the end of the movement (the last time the tangential velocity was greater than 5% of the peak tangential velocity). Reaction time (s) was defined as the time between when the target appeared in green, and movement onset (as defined above). Endpoint error (cm) was defined as the straight-line distance from the stylus location at the end of the movement to the center of the target (cm). Improved motor performance was indicated by a shorter movement time, a shorter reaction time and a smaller endpoint error.

### Sensation tasks

TPOD: This task involved presentation of a two-pronged instrument (spacing 5, 4, 3 and 2 mm) to the palmar side of the distal pads of the left index and little fingers (Fig. 1b2). These prongs were oriented randomly either across or down (trial-by-trial), with respect to the proximal–distal finger axis, using descending spacing order (see^41^ for advantages of this task over the traditional two-point discrimination task). Initially, the participants were required to indicate each orientation of the prongs to the palmar pad of the middle finger with their eyes open and closed to familiarize themselves with the task. Then, participants verbally indicated the orientation at each trial with their eyes closed. Proportion of correct trials were averaged as a measure of tactile spatial acuity^41^.

Proprioception: The participants performed a point-to-point movement task on the same graphics tablet that was used for the motor task. The task is a version of a similar, previously used paper and pencil task—the Brief Kinesthesia Test^42^, in which the participant was asked to actively reproduce reaching movements without vision after being passively guided by the examiner. The passive movement of the participant’s arm was performed here using a custom-made device, which was connected to the tablet (Fig. 1b3). The linear actuator of the device was designed as a toothed-belt-driven carriage, sliding on two circular steel rods of diameter 10 mm. It was actuated by a NEMA-17 stepper motor, controlled by an Arduino Uno controller and a DRV8825 driver. The resolution of the movement is 50 pulses to mm, i.e., it provides a high accuracy of movement. At the beginning of every actuation, a homing was done by a microswitch located at the starting position. As a preliminary step for using the linear actuator, we tested it to make sure it could withstand the required loads. It can carry more than 20 kg without slipping or velocity reduction.

Initially, to familiarize the participants with the task, they were required to hold the stylus, without the device, in their left hand and reproduce a reaching movement from the starting position to target 3 after being passively moved by the examiner’s hand, with their eyes open and eyes closed. Then, with eyes closed and blindfolded, the assessment with the device began. The non-dominant left arm of the participant that held the stylus was placed on the device. After holding the stylus at the starting point, the device passively moved the arm, from the starting point to target 2, 17 cm distant from the starting point, over two sec. After remaining at target 2 for 500 ms, the tablet’s screen changed its color from black to white, and the examiner lifted the participant’s arm from the device and returned it to the starting position to allow the participant to actively reproduce the reaching movement, without the device. The participants were instructed to reach toward the target and stay there. After 500 ms, the tablet’s screen changed its color from black to white, and the examiner lifted the participant’s arm and returned it to the starting position for the next passive reaching movement. The pretest and the posttest consisted of 10 passive and 10 active reaching movements. Two outcome measures for the proprioception were used: movement time (s) of the reaching movements and endpoint error (cm) (see the section of motor sequence learning task).

Trials that were not properly recorded by the tablet for the motor and proprioception tasks were discarded.

### Statistical analysis

Age and sex were compared between groups (S1, M1, sham) using Kruskal–Wallis (as age was not normally distributed) and chi-squared tests, respectively. Differences between groups with respect to each of the outcome measures in the pretest were investigated using one-way analysis of variance (ANOVA) with Bonferroni correction for multiple comparisons. The effects of stimulation, sex and time on the outcome measures were investigated using a mixed design ANOVA with time (pretest, posttest) as the within-subject factor, and group (S1, M1, sham) and sex (male, female) as the between-subject factors with Bonferroni correction for multiple comparisons. Since reaction time values were not normally distributed in each group, they were log-transformed before this analysis (the original values are presented for clarity). The differences between groups with respect to the frequency of adverse effects were investigated using a chi-squared test. The differences between groups with respect to the discomfort from adverse effects were investigated using Kruskal–Wallis with Bonferroni correction for multiple comparisons [for a similar approach see^43^]. In addition, due to an imbalanced number of males and females within the groups, and low number of participants in each group, a mixed model ANCOVA was conducted with time as the within-subject factor, group as the between-subject factors and sex as covariate with Bonferroni correction for multiple comparisons. All tests were done using SPSS (version 26.0) with initial significance levels of *p* < 0.05.

## Results

**Figure 3:**
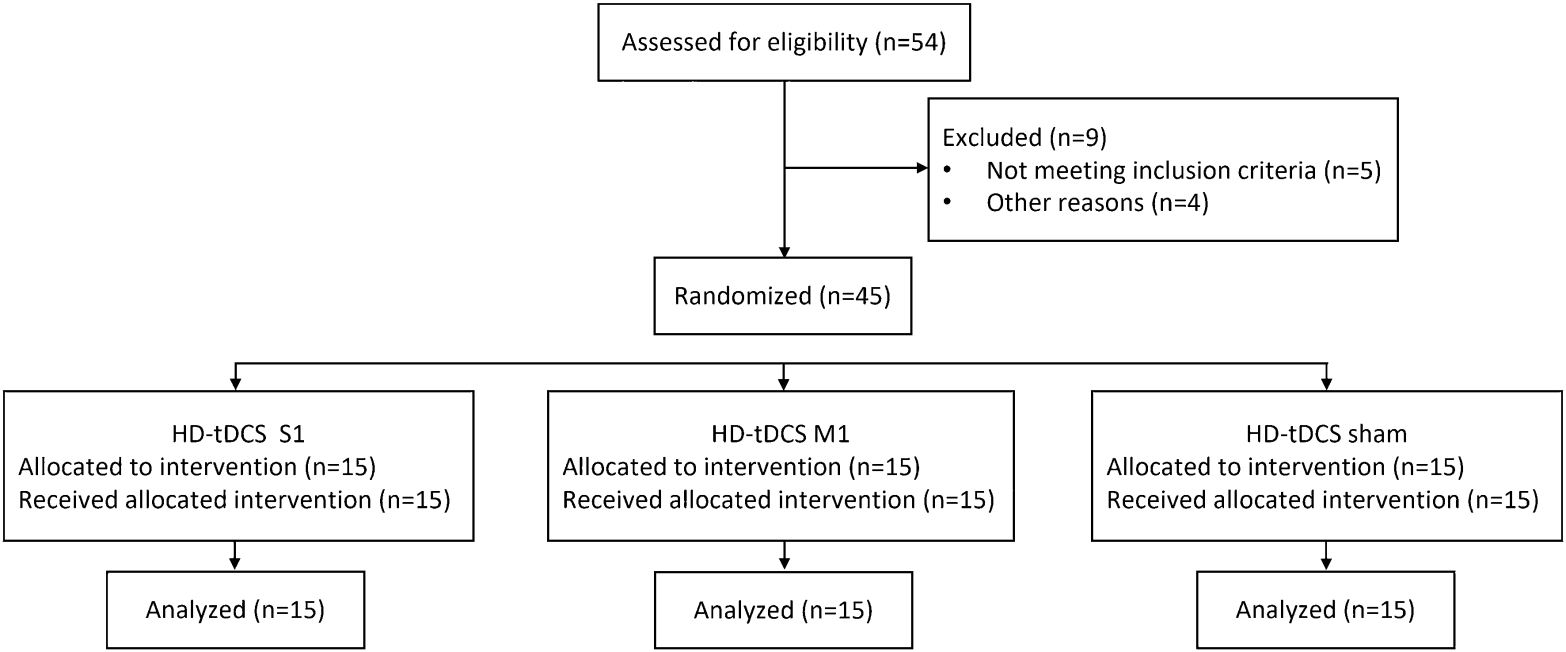
Trial flowchart. HD-tDCS S1/M1 = High-definition transcranial direct current stimulation of primary somatosensory cortex (S1)/primary motor cortex (M1).

The flowchart illustrating the process of the study is shown in Fig. 3. Fifty-four participants underwent the pre-enrollment screening evaluation. Of those, nine did not meet inclusion criteria. Age (S1 group: 23.5 ± 2.6 years; M1 group: 23.9 ± 2.6 years; sham group: 23.3 ± 1.8 years) and sex (S1 group: six women; M1 group: eight women; sham group: seven women) did not differ between groups (*p* > 0.717, for all).

### Motor sequence learning task

Mean values of movement time (s), reaction time (s) and endpoint error (cm) by group and time are shown in Table 1. All these outcome measures did not show significant differences between groups in the pretest (*p* > 0.508, for all).

**Table 1:** Means, standard deviations and confidence intervals of reaction time, movement time and endpoint error of the motor task for stimulation groups in time points.

Variable	S1 group (n = 15)		M1 group (n = 15)		Sham group (n = 15)	
	Pretest	Posttest	Pretest	Posttest	Pretest	Posttest
Reaction time (s): Mean ± SD [*CI]	0.40 ± 0.10 [0.35–0.46]	0.38 ± 0.11 [0.32–0.44]	0.41 ± 0.13 [0.34–0.49]	0.40 ± 0.11 [0.34–0.46]	0.46 ± 0.18 [0.36–0.56]	0.46 ± 0.25 [0.32–0.59]
Movement time (s): Mean ± SD [*CI]	0.84 ± 0.22 [0.72–0.96]	0.79 ± 0.18 [0.67–0.89]	0.79 ± 0.24 [0.66–0.92]	0.74 ± 0.19 [0.64–0.85]	0.88 ± 0.37 [0.68–1.09]	0.79 ± 0.29 [0.63–0.94]
Endpoint error (cm): Mean ± SD [*CI]	0.36 ± 0.10 [0.30–0.42]	0.34 ± 0.09 [0.29–0.39]	0.43 ± 0.16 [0.33–0.52]	0.37 ± 0.10 [0.31–0.42]	0.41 ± 0.18 [0.31–0.51]	0.39 ± 0.12 [0.32–0.45]

S1/M1 group: high-definition transcranial direct current stimulation over S1/M1, respectively. *CI = 95% confidence interval.

#### Effects on reaction time (s)

**Figure 4:**
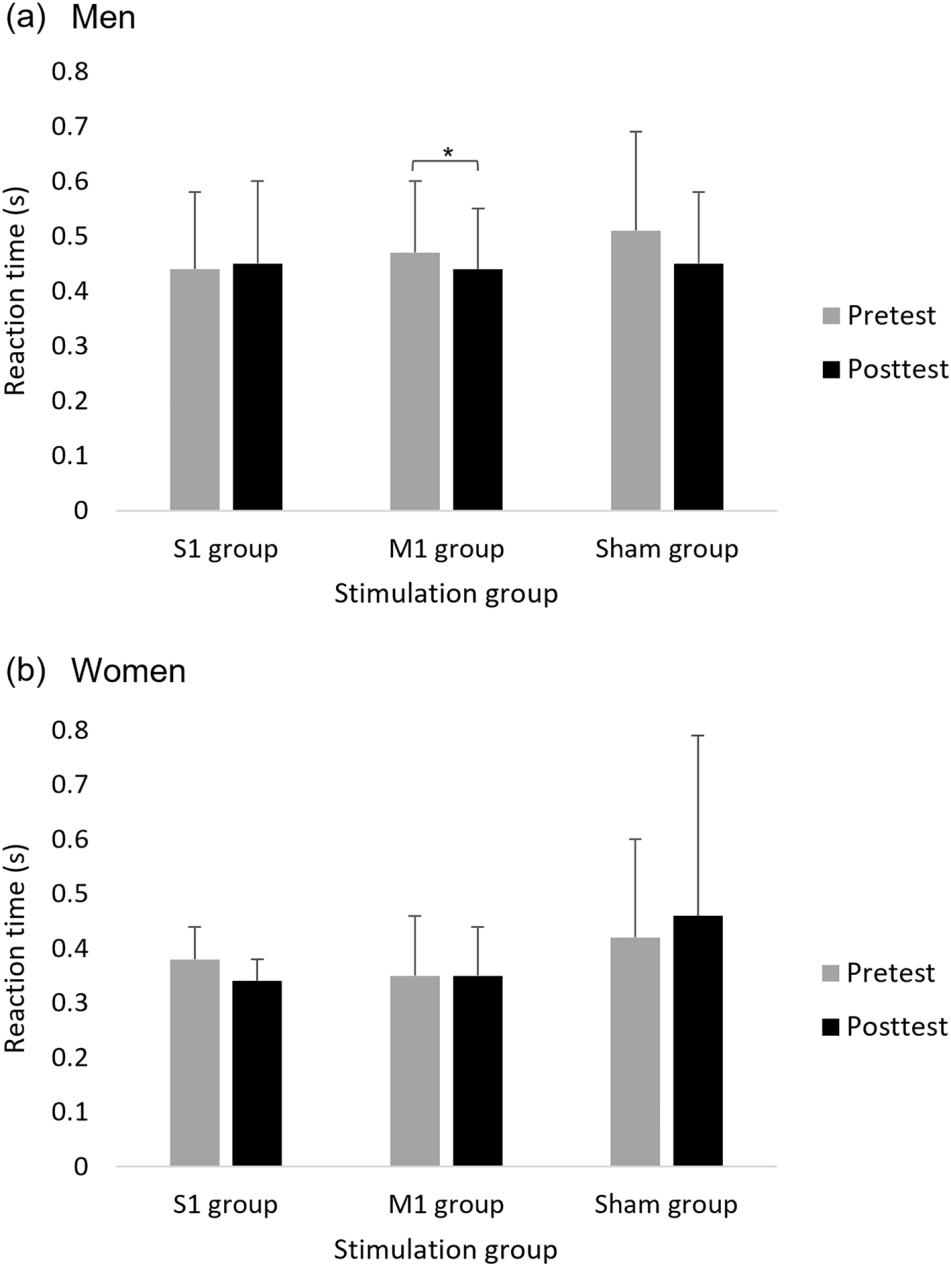
Mean reaction time (s) of reaching movements in pretest and posttest in the three groups in men (**a**) versus women (**b**). Error bars show standard deviation. Asterisks denote a significant difference.

A main effect of Sex (F(1,39) = 5.206; *p* = 0.028; partial η2 = 0.12; observed power = 0.61) showed that, overall, reaction time was faster in women (0.38 ± 0.27 s) compared to men (0.46 ± 0.12 s). Group x Time interaction varied across Sex as shown by a second-order interaction between Sex x Group x Time (F(2,39) = 4.419; *p* = 0.019; partial η2 = 0.19; observed power = 0.73). Only in men, the interaction Group x Time was significant (F(2,18) = 3.914; *p* = 0.039; partial η2 = 0.30; observed power = 0.63) such that only in M1 group, reaction time decreased significantly in posttest (0.44 ± 0.11 s) compared to pretest (0.47 ± 0.13 s) (F(1,7) = 15.109; *p* = 0.006; partial η2 = 0.68; observed power = 0.91) (Fig. 4). No other significant effects were observed.

#### Effects on movement time (s)

**Figure 5:**
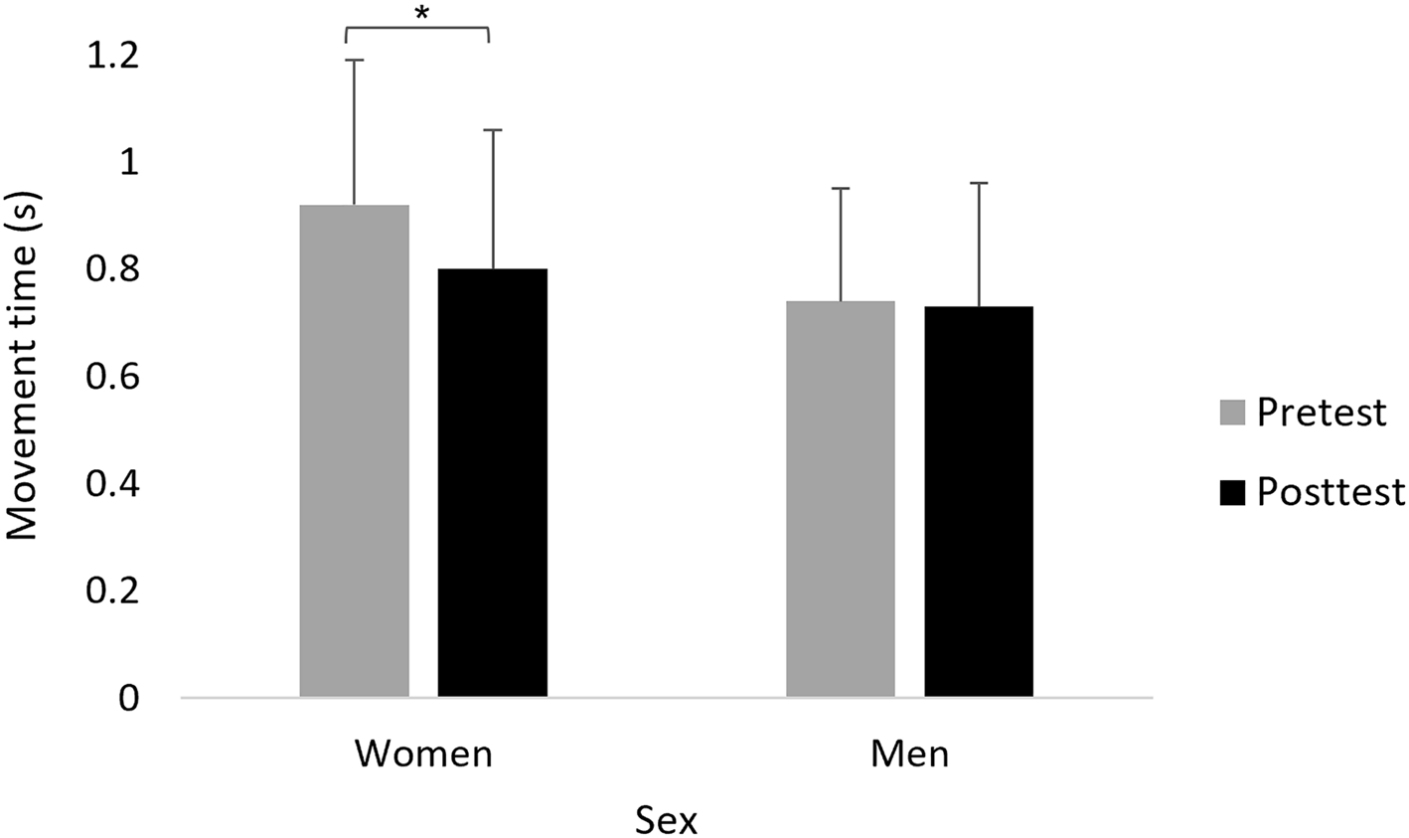
Mean movement time (s) of reaching movements in women and men in pretest and posttest, across stimulation groups (collapse across stimulation groups is presented because the interaction Time × Sex × Group was not significant). Error bars show standard deviation. Asterisks denote a significant difference.

A main effect of Time (F(1,39) = 13.550; *p* = 0.001; partial η2 = 0.26; observed power = 0.95) showed that, overall, movement time decreased significantly in posttest (0.77 ± 0.22 s) compared to the pretest (0.83 ± 0.28 s). This effect was, however, modulated by Sex, as was shown by the interaction of Sex x Time (F(1,39) = 12.460, *p* = 0.001; partial η2 = 0.24; observed power = 0.93). Only in women, movement time decreased significantly in posttest (0.80 ± 0.21 s) compared to the pretest (0.92 ± 0.27 s), across groups (F(1,21) = 21.210, *p* < 0.001; partial η2 = 0.51; observed power = 0.99) (Fig. 5). No other significant effects were observed.

#### Effects on endpoint error (cm)

**Figure 6:**
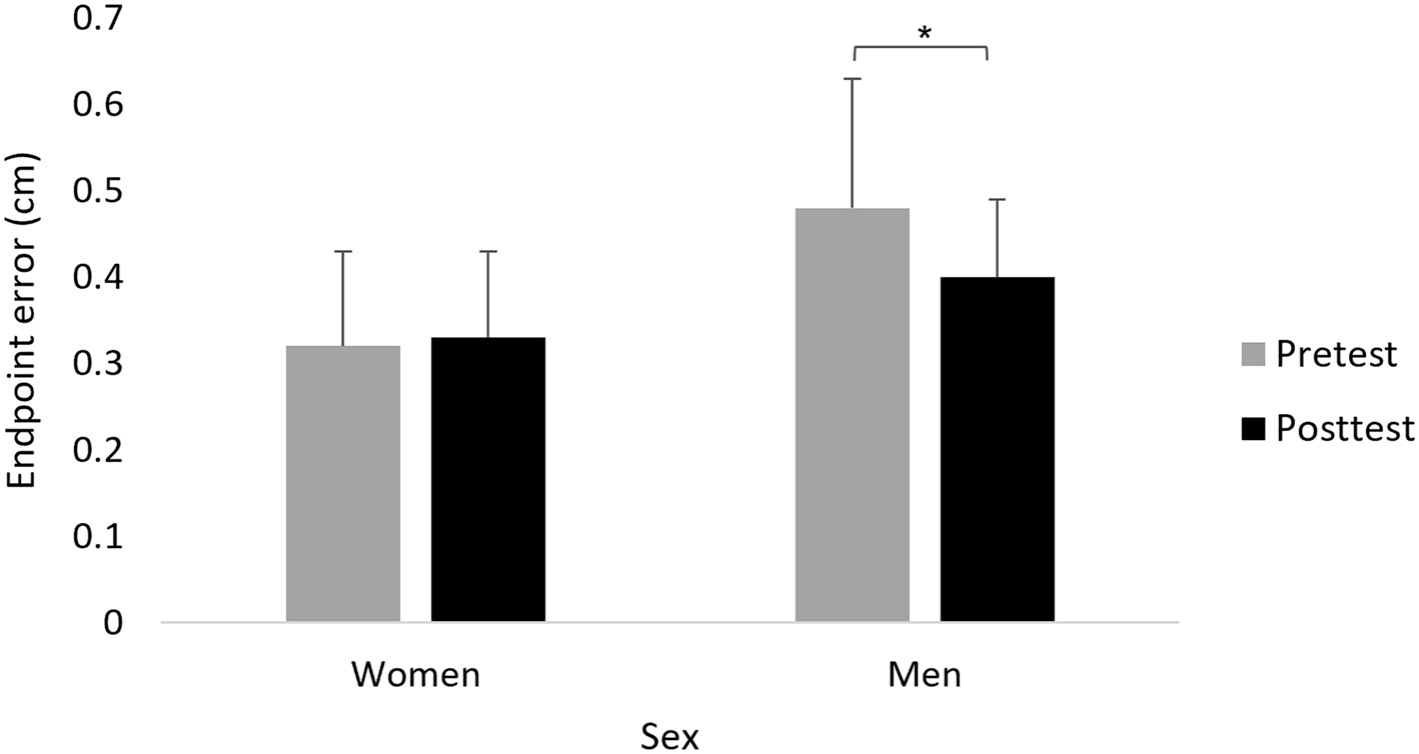
Mean endpoint error (cm) of reaching movements in women and men in pretest and posttest, across stimulation groups (collapse across stimulation groups is presented because the interaction Time × Sex × Group was not significant). Error bars show standard deviation. Asterisks denote a significant difference.

A main effect of Sex (F(1,39) = 14.005; *p* = 0.001; partial η2 = 0.26; observed power = 0.95) showed that, overall, endpoint error was smaller in women (0.33 ± 0.10 cm) compared to men (0.44 ± 0.13 cm). A main effect of Time (F(1,39) = 4.483; *p* = 0.041; partial η2 = 0.10; observed power = 0.54) showed that, overall, endpoint error was smaller in posttest (0.37 ± 0.10 cm) compared to pretest (0.40 ± 0.15 cm). This effect was, however, modulated by Sex, as was shown by the interaction of Sex x Time (F(1,39) = 6.335; *p* = 0.016; partial η2 = 0.14; observed power = 0.69). Only in men, the endpoint error was significantly smaller in posttest (0.40 ± 0.09 cm) compared to pretest (0.48 ± 0.15 cm) (F(1,18) = 9.938; *p* = 0.006; partial η2 = 9.94; observed power = 0.85) (Fig. 6). No other significant effects were observed.

### Sensation tasks

Mean values of proportion of correct trials in the TPOD task, and movement time (s) and endpoint error (cm) in the proprioception task by group and time are shown in Table 2. All these outcome measures did not differ between groups in the pretest (*p* > 0.360, for all).

**Table 2:** Means, standard deviations and confidence intervals of TPOD, movement time and endpoint error of the sensation tasks for stimulation groups in time points.

Variable	S1 group (n = 15)		M1 group (n = 15)		Sham group (n = 15)	
	Pretest	Posttest	Pretest	Posttest	Pretest	Posttest
TPOD (%): Mean ± SD [*CI]	70.31 ± 12.90 [64.11–78.39]	81.25 ± 11.81 [74.71–87.79]	64.17 ± 12.60 [57.19–71.15]	65.83 ± 16.34 [56.78–74.88]	67.08 ± 14.84 [58.87–75.30]	70.00 ± 11.38 [63.70–76.30]
Movement time (s): Mean ± SD [*CI]	2.25 ± 0.84 [1.78–2.72]	1.94 ± 0.71 [1.55–2.33]	2.28 ± 0.44 [2.04–2.52]	2.00 ± 0.46 [1.75–2.26]	2.24 ± 0.79 [1.80–2.68]	2.00 ± 0.73 [1.59–2.40]
Endpoint error (cm): Mean ± SD [*CI]	3.33 ± 1.23 [2.66–4.02]	3.16 ± 1.43 [2.37–3.96]	2.97 ± 1.42 [2.19–3.75]	2.81 ± 0.96 [2.27–3.34]	3.00 ± 1.55 [2.14–3.86]	3.16 ± 1.26 [2.47–3.86]

TPOD = Two-point orientation discrimination; S1/M1 group: high-definition transcranial direct current stimulation over S1/M1, respectively. *CI = 95% confidence interval.

### Two-point orientation discrimination

**Figure 7:**
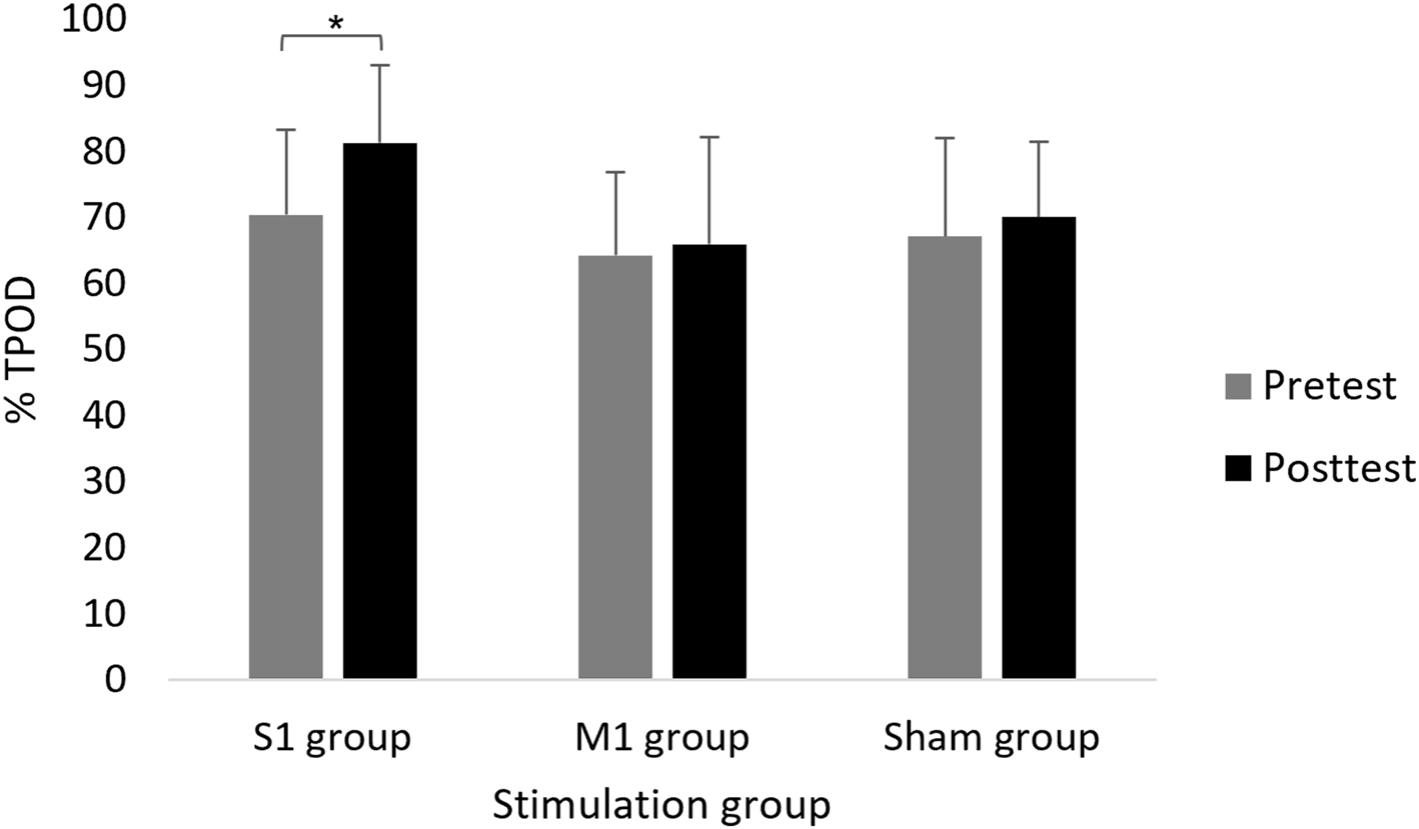
Mean percentage of correct answers in the two-point orientation discrimination (TPOD) test in each group at pretest and posttest. Error bars show standard deviation. Asterisks denote a significant difference.

A main effect of Time (F(1,39) = 8.149; *p* = 0.007; partial η2 = 0.17; observed power = 0.80) showed that, overall, the percent of correct trials was higher in posttest (72.28 ± 14.63%) compared to pretest (67.23 ± 13.50%). The interaction of Group x Time reached border-line significance (F(2,39) = 2.765; *p* = 0.075; partial η2 = 0.12; observed power = 0.51). Our interest was focused on clarifying whether the proportion of correct trials differed between groups at timepoints and whether the proportion of correct trials differed between time points within each group. Therefore, despite the borderline significance, the interaction was further investigated. Only in the S1 group, the proportion of correct trials increased significantly in posttest (81.25 ± 11.81%) compared to pretest (70.31 ± 12.90%) (F(1,13) = 8.493; *p* = 0.012; partial η2 = 0.40; observed power = 0.77) (Fig. 7). The effect size of the change in the correct trials from pretest to posttest was high for S1 (D = 0.7) whereas it was low for M1 group (D = 0.2) and sham group (D = 0.3). In addition, only in posttest, the proportion of correct trials differed between groups (F(2,42) = 5.339; *p* = 0.009) such that it was significantly higher in the S1 group compared to the M1 group (65.83 ± 16.34%; pBonferroni = 0.009), and just tended to be higher compared to the sham group (70.00 ± 11.38%; *p* = 0.026; pBonferroni = 0.079). No other significant effects were observed.

### Proprioception

#### Effects on movement time (s)

A main effect of Time (F(1,39) = 26.688; *p* < 0.001; partial η2 = 0.41; observed power = 0.99) showed that, overall, movement time was shorter in posttest (1.97 ± 0.63 s) compared to pretest (2.24 ± 0.70 s). No other significant effects were observed.

#### Effects on endpoint error (s)

No significant effects were found.

### Adverse effects

The stimulation was well tolerated by the participants, and no sessions were aborted due to adverse effects. The occurrence of adverse effects in the S1, M1 and sham groups are displayed in Table 3. Frequency of adverse effects and discomfort from the adverse did not differ between the groups.

**Table 3:** Frequency and discomfort of adverse effects.

Adverse effect	S1 group (n = 15)		M1 group (n = 14*)		Sham group (n = 15)	
	Frequency	Discomfort	Frequency	Discomfort	Frequency	Discomfort
Tingling	(67%) 10	2 (0–4)	8 (57%)	3.5 (0–6)	(60%) 9	3 (0–6)
Burning	(13%) 2	0 (0–0)	(29%) 4	0 (0–3.5)	(20%) 3	0 (0–0)
Itching	(40%) 6	0 (0–7)	(43%) 6	0 (0–6.25)	(47%) 7	0 (0–5)
Headache	0 (0%)	0 (0–0)	1 (7%)	0 (0–0)	0 (0%)	0 (0–0)

S1/M1 group: high-definition transcranial direct current stimulation over S1/M1, respectively. Median values and interquartile ranges of discomfort are presented. *Data from one subject were not included due to technical loss of information.

The mixed design ANOVA effects for motor and sensation tasks are displayed in detail in Supplementary Table 1. The ANCOVA results for motor and sensation tasks are displayed in detail in Supplementary Table 2.

## Discussion

To the best of our knowledge, this is the first study that evaluated the effects of tDCS over S1 on motor performance in adults. In addition, we considered the sex-mediated effects of the stimulation on the response. By means of HD-tDCS applied with optimized electrode configurations for maximal focal active stimulation, a considerably nuanced determination of stimulation site (S1, M1, and sham stimulation) effects on motor performance was achieved. We also investigated the effects on sensation perception, as a secondary outcome measure. We found that reaction time, movement time, and endpoint error of a sequence of reaching movements did not differ between S1, M1, and sham groups. Nonetheless, reaction time improved from pretest to posttest in the men from the M1 group only. Reaching movement time improved from pretest to posttest in women only, in a similar manner in all stimulation groups, whereas endpoint error improved in men only. Regarding sensation perception, percent of correct trials for the TPOD was significantly higher in the S1 group compared to the M1 group in the posttest (but not in the pretest), and improved from pretest to posttest in the S1 group only. The reaching movement time of the proprioception task improved from pretest to posttest, in a similar manner in all groups, and endpoint error did not change.

Our finding that HD-tDCS over S1 did not improve motor performance as compared to sham stimulation in healthy participants is not in line with our first hypothesis. We had assumed positive effects in motor performance for the S1 stimulation based on the neuroanatomical^1^ and behavioral links^8,11,29,38^ between the sensory and motor systems. Improved motor performance was found following tDCS over sensory areas in rats^29^, and following TMS over S1 in both healthy participants^38^ and individuals with stroke^8,11^. Exciting ipsilesional S1 using 5 Hz rTMS with skilled motor practice enhanced motor learning in individuals with chronic stroke^8^. Meehan et al.^11^ found that continuous theta burst stimulation over contralesional S1 or M1 in individuals with chronic stroke enhanced performance of serial targeting tasks performed by the hemiparetic upper limb. They even found that stimulation over contralesional S1 led to significant changes in the functional ability of the hemiparetic upper limb, indexed by time to complete the Wolf Motor Function Test, as compared to stimulation over the contralesional M1, or control stimulation. The different mechanisms underlying the effects of tDCS versus TMS may partly explain the more potent effects of TMS than those of tDCS. In contrast to TMS, which directly induces action potentials^71^, tDCS alters spontaneous brain activity and excitability by modulating the neuronal membranes in a polarity dependent manner^20^. It is also possible that the stimulation protocol of the S1 group in the current study is not optimal for enhancing tDCS effects on motor performance. Variable and even inverted neurophysiological^15,21–24^ and behavioral effects^25,26^ were found in response to different tDCS protocols, depending on several parameters such as stimulation intensity and duration. For example, conventional anodal stimulation at 2 mA led to faster reaction times than cathodal stimulation, but only before 13 min of stimulation had elapsed, whereas after 13 min, the reaction times under cathodal stimulation were faster^72^. However, the optimal protocol is yet to be determined. On the other hand, it should be noted that, as with our results, in some TMS studies, stimulating S1 in healthy participants^73^ and those with stroke^74^ did not improve motor performance either. The mixed results in the non-invasive neuromodulation studies may be explained by the different protocols (such as rTMS vs. theta burst TMS), the outcome measures used, and variable responses in humans to non-invasive brain stimulation techniques, e.g., due to differences in the individual morphology of the cerebrospinal fluid and brain^21,75^.

Whereas HD-tDCS over S1 in the current experimental setup did not improve motor performance, it did improve the percent of correct trials for the TPOD from pretest to posttest; moreover, the percent of correct trials was significantly higher in the S1 group compared to the M1 group in the posttest (but not in the pretest). The percent of correct trials in the S1 group was not significantly higher compared to the sham group (*p* = 0.026; pBonferroni = 0.079). However, the effect sizes of the change between pretest and posttest were high in the S1 group (D = 0.7) but low in the sham and M1 groups (D = 0.3 and D = 0.2, respectively). These results are reasonably in line with our second hypothesis. Given S1’s essential role in somatosensory processing, it is reasonable to assume that stimulating S1 would produce more effects than the sham and M1 stimulations on sensation perception. The modulatory excitatory and inhibitory effects that are exerted by M1 on S1 via anatomical projections^1,76,77^ may have also contributed to sensorimotor integration and to the augmented effects in sensation perception specifically after S1 stimulation because simultaneously to the S1 stimulation, the participants performed 18 sequences of the motor task. Our results are also consistent with previous findings in which healthy participants^30,33^ and those with stroke^31^ improved their performance of the tactile spatial discrimination task following active tDCS with an anodal electrode placed over the S1. However, it should be noted that, as mentioned in the methods and results section, a mixed model ANCOVA was also conducted (with sex as covariate) due to an imbalanced number of males and females within the groups, and low number of participants in each group. In the ANCOVA results (Supplementary Table 2), the percent of correct trials only tended to be higher in the S1 group compared to the M1 group in the posttest (*p* = 0.068). Therefore, further studies with larger sample sizes are required for validation of the current results.

Even though tDCS can modulate the proprioceptive afferent system by changing the excitability of projections to propriospinal neurons^78,79^, HD-tDCS over S1 did not improve proprioception in the current study. Muffel et al.^32^ found that active tDCS with an anodal electrode placed over S1 elicited opposing effects on proprioceptive accuracy as a function of age. While young adults (27.0 ± 2.4 years) showed a slight improvement, older adults (69.4 ± 4.9 years) exhibited a decline in performance during a-tDCS. This study, similar to our own, used 1 mA tDCS for 15 min; however, in contrast to our study, Muffel et al.^32^ used a different experimental design (crossover), other assessments of somatosensory perception (an arm position matching task in a robotic environment), and a different tDCS montage (conventional). Taken together, these differences could explain the contradictory results. It is also important to note that the current simulation used in the Muffel et al. study^32^ showed a non-focal distribution of the induced electric field throughout the sensorimotor system. In light of the interhemispheric interactions between S1 and the secondary somatosensory cortex (S2) in humans^80,81^, and the involvement of S2 in sensation perception^82^, the larger spread of the current in the Muffel et al. study^32^ could have led to the improved proprioception in young adults. Indeed, sensation perception improved following tDCS over S2 compared to sham stimulation in individuals with stroke^31^. Our finding that reaching movement time for the proprioception task improved from pretest to posttest similarly in all groups can be related to the initial fast learning phase during the session^83^. Interestingly, such an initial fast learning phase did not occur in any group for the endpoint error of the reaching movement in the proprioception task. This may reflect the complexity of performing a reaching movement toward the center of the target with eyes closed. It is possible that additional repetitions would have improved the endpoint error.

Sex modulated the effects of active HD-tDCS over M1 on reaction time, which improved from pretest to posttest only in the men in the M1 group. Similarly, Gorbet and Stains^52^ found that reaction time (but not movement time) slowed in men but not in women following inhibitory continuous theta burst stimulation (using TMS) over the contralateral dorsal premotor cortex during visually guided reaching movements, a task similar to the one used in the current study. In the Gorbet and Stains study^52^ and in ours, the specific influence of non-invasive brain stimulation on reaction time in men may be related to previous findings, which pointed to more bilateral patterns of activity in women and unilateral activity in men in different brain regions during motor tasks^84^ and others, such as mental rotation^85^. If men rely more on the contralateral hemisphere, they might be more responsive than women to stimulation in that hemisphere. The finding of sex differences in reaction time but not movement time in the Gorbet and Stains study^52^ led them to suggest that sex differences in visually guided reaching movements are probably more strongly associated with movement planning than with motor execution. This suggestion may explain our finding that only reaction time and not movement time improved following HD-tDCS in men.

Additional reasons for sex-related differences in the responses to tDCS may be related to hormonal differences and bone density. Hormonal levels fluctuate significantly more in women than in men^56,86^. As it appears that progesterone drives the increase of cortical inhibition and estradiol enhances excitability^56–58^, it is possible that the phase of menstrual cycle of the women that participated in the current study affected their responsiveness to the tDCS. However, we did not control for the menstrual phase. With regard to bone density, a computational study indicated that the induced electric field is higher in female head models, on average, than male head models across several metrics^48^. By contrast, in other tDCS studies, men were found to receive more current than women^47,87,88^. In addition, men were found to have a more cancellous parietal bone and females a denser parietal bone. Indeed, studies have indicated sex-related anatomical differences in head structures^89–91^. Our finding supports the evidence that sex moderates the effects of tDCS^51–55^. It seems that the high rates of inter- and intra-individual variability with regard to the effects of tDCS on motor performance^92^ may also be related to sex^56^. tDCS studies may find more meaningful results if they are analyzed according to sex.

The improvement in perception discrimination but not motor performance following HD-tDCS over S1, and the improvement in reaction time but not sensation perception in the men from the M1 group may point to a selective HD-tDCS influence that is dependent on the stimulation site. Despite reciprocal projections between S1 and M1, it seems that this input is relatively weak with regards to producing behavioral effects. Indeed, Kinnischtzkeet al.^77^ found that M1 provides input to nearly all S1 pyramidal neurons in the mouse, yet most of that input on its own was unlikely to make S1 neurons fire. It seems that using HD-tDCS^44,46,53^ together with the brain modelling software to determine the tDCS montage for maximal focal stimulation of the right S1 and M1 improved the spatial focality of the current and induced stimulation site effects. On the other hand, since the measures of the motor reaching sequence task did not differ between groups, and the TPOD that differed between the S1 and M1 group only tended to do so between the S1 and sham group, it should be acknowledged that this interpretation relates to moderate effects that were mainly found within groups and not between groups.

We also found sex-mediated effects that are not related specifically to the stimulation type. For the motor task, reaching movement time improved from pretest to posttest in a similar manner in all stimulation groups, only in women, whereas reaching endpoint error improvement was found only in men. This finding is in line with previous evidence regarding differences in motor ability between the sexes^93–97^. Similar to the specific improvement found here in reaching reaction time in women, females also perform the 9-hole peg test, a measure of finger dexterity, faster than males^94,95^. There is a biological basis for sex differences^98^, which also encompasses the motor domain, for example, female rats have 20% fewer dopaminergic neurons which are involved in motor control^99^. The similar improvement in movement time and endpoint error from pretest to posttest across groups, including sham stimulation, in women and men, respectively, may reflect motor learning over time and a placebo effect. The motor learning over time can be related to the initial fast-learning phase within session^83^. The possible placebo effect could be related to the finding that the frequency of adverse effects and discomfort from the adverse did not differ between the groups.

The study has several limitations. First, despite improved spatial focality of current using HD-tDCS and brain modelling software to determine the tDCS montage for maximal focal stimulation of S1 and M1, one cannot assume that stimulation was exclusively delivered to S1 or M1, and the electric fields (V/m) could have differed between groups because of differences in the individual participants’ anatomical features^100^. Responses to the HD-tDCS may also be related to variable anatomical features. Second, as the current study design was a single-blind randomized controlled study, the experimenter was not blinded to group allocation. It should be noted, though, that besides the TPOD task which was based on the experimenters’ scoring (based on a structured protocol), the scoring of the motor and proprioception task was automatically computed by the MATLAB software. Third, because changes in the levels of estradiol and progesterone during the follicular and luteal phases affect cortical excitation/inhibition and behavior^50–58^, it is possible that not recruiting women in the same phase of the menstrual cycle increased the variability of the tDCS related effects on motor performance and sensation perception. For example, it is possible that women who are tested in the second half of the follicular phase which is characterized by high level of cortical excitation^86^ may respond better to the tDCS. Fourth, we did not take into consideration the smoking habits of the participants. This potentially confounding factor could have affected the results because nicotine may affect cortical excitability^101^ and tDCS effects on MEPs^102^. Fifth, the montage of the sham group was similar to the montage of the S1 group only. It may have been useful to add a sham group with a montage that is similar to the M1 group or alternatively to randomize the montage of the sham group to be similar to either the S1 group or M1 group. It should be noted, though, that the frequency of adverse effects and discomfort from the adverse did not differ between the groups. Lastly, despite sample size calculation, the value of power and effect size (partial η2) was smaller than 0.8 and 0.14, respectively (these values are equivalent to acceptable power and high effect size), for some of the results (Supplementary Table 1), and number of males and females was not equal in each of the groups. Therefore, the findings reported here should be reproduced in larger cohorts.

Future tDCS studies should consider menstrual phases of women to control for the hormonal fluctuations and their effect on cortical excitation and behavioral effects^56,86^. Men lack the cyclic fluctuation of sex hormones^56^; however, the relative contribution of testosterone may also be taken into consideration. Hormonal levels and bone composition of each individual should be measured. As sex differences are founded in biological variants inherent in men and women^103^, it is important to take into account the participants’ biological sex when analyzing effects of tDCS.

## Conclusions

A montage of 15-min active HD-tDCS at 1 mA with focal stimulation of the primary sensory cortex did not affect motor performance but did improve tactile discrimination perception in young healthy participants. Such stimulation over M1 improved reaction time in men only but did not affect movement time and endpoint error of reaching movements or sensation perception. These findings suggest that despite the links between the sensory and motor systems, the effect of excitatory stimulation of S1 and M1, at least in the current montages, may be more specific for influencing sensation perception and motor performance, respectively, i.e., stimulation focality matters. In addition, the results demonstrate sex-mediating effects of HD-tDCS on motor performance. The influence of stimulation site and sex should be taken into consideration in clinical tDCS studies that aim to improve upper limb sensorimotor functioning in individuals with stroke.

## Supplementary Information

Supplementary Table 1.

Supplementary Table 2.

## Data Availability

The datasets generated and/or analysed during the current study are available in the Figshare repository, https://figshare.com/account/home#/data.

## References

[1] Edwards L, King EM, Buetefisch C, Borich M (2019). Putting the “sensory” into sensorimotor control: The role of sensorimotor integration in goal-directed hand movements after stroke. Front. Integr. Neurosci..

[2] Meyer S, Karttunen AH, Thijs V, Feys H, Verheyden G (2014). How do somatosensory deficits in the arm and hand relate to upper limb impairment, activity, and participation problems after stroke? A systematic review. Phys. Ther..

[3] Pavlides C, Miyashita E, Asanuma H (1993). Projection from the sensory to the motor cortex is important in learning motor skills in the monkey. J. Neurophysiol..

[4] Pipereit K, Bock O, Vercher JL (2006). The contribution of proprioceptive feedback to sensorimotor adaptation. Exp. Brain. Res..

[5] Rand D (2018). Proprioception deficits in chronic stroke-Upper extremity function and daily living. PLoS ONE.

[6] Rothwell JC (1982). Manual motor performance in a deafferented man. Brain.

[7] Tuthill JC, Azim E (2018). Proprioception. Curr. Biol..

[8] Brodie SM, Meehan S, Borich MR, Boyd LA (2014). 5 Hz repetitive transcranial magnetic stimulation over the ipsilesional sensory cortex enhances motor learning after stroke. Front. Hum. Neurosci..

[9] Debas K, al. (2010). Brain plasticity related to the consolidation of motor sequence learning and motor adaptation. Proc. Natl. Acad. Sci. USA.

[10] Hamdy S, Rothwell JC, Aziz Q, Singh KD, Thompson DG (1998). Long-term reorganization of human motor cortex driven by short-term sensory stimulation. Nat. Neurosci..

[11] Meehan SK, Dao E, Linsdell MA, Boyd LA (2011). Continuous theta burst stimulation over the contralesional sensory and motor cortex enhances motor learning post-stroke. Neurosci. Lett..

[12] Vidoni ED, Acerra NE, Dao E, Meehan SK, Boyd LA (2010). Role of the primary somatosensory cortex in motor learning: An rTMS study. Neurobiol. Learn. Mem..

[13] Snell, R. S. Corticospinal tracts. In *Clinical Neuroanatomy*. 155, 7th ed. (Wolters Kluwer Health/Lippincott Williams & Wilkins, 2010).

[14] Calautti C (2007). The relationship between motor deficit and hemisphere activation balance after stroke: A 3T fMRI study. Neuromiage.

[15] Nitsche MA, Paulus W (2000). Excitability changes induced in the human motor cortex weak transcranial direct current stimulation. J. Physiol..

[16] Patel R (2019). The impact of transcranial direct current stimulation on upper-limb motor performance in healthy adults: A systematic review and meta-analysis. Front. Neurosci..

[17] Broeder S (2015). Transcranial direct current stimulation in parkinson’s disease: Neurophysiological mechanisms and behavioral effects. Neurosci. Biobehav. Rev..

[18] Kang N, Summers JJ, Cauraugh JH (2016). Transcranial direct current stimulation facilitates motor learning post-stroke: A systematic review and meta-analysis. J. Neurol. Neurosurg. Psychiatry..

[19] Sánchez-Kuhn A, Pérez-Fernández C, Cánovas R, Flores P, Sánchez-Santed F (2017). Transcranial direct current stimulation as a motor neurorehabilitation tool: An empirical review. Biomed. Eng. Online.

[20] Stagg CJ, Antal A, Nitsche MA (2018). Physiology of transcranial direct current stimulation. J. ECT..

[21] Ammann C, Lindquist MA, Celnik PA (2017). Response variability of different anodal transcranial direct current stiomulation intensities across multiple sessions. Brain stimul..

[22] Batsikadze G, Moliadze V, Paulus W, Kuo MF, Nitsche MA (2013). Partially non-linear stimulation intensity-dependent effects of direct current stimulation on motor cortex excitability in humans. J. Physiol..

[23] Moliadze V (2015). Stimulation intensities of transcranial direct current stimulation have to be adjusted in children and adolescents. Clin. Neurophysiol..

[24] Strube W (2016). Bidirectional variability in motor cortex excitability modulation following 1 mA transcranial direct current stimulation in healthy participants. Physiol. Rep..

[25] Lerner O, Friedman J, Frenkel-Toledo S (2021). The effect of high-definition transcranial direct current stimulation intensity on motor performance in healthy adults: A randomized controlled trial. J. Neuroeng. Rehabil..

[26] Ehrhardt SE, Filmer HL, Wards Y, Mattingley JB, Dux PE (2021). The infuence of tDCS intensity on decision-making training and transfer outcomes. J. Neurophysiol..

[27] Gauthier LV (2008). Remodeling the brain plastic structural brain changes produced by different motor therapies after stroke. Stroke.

[28] Ko SB, Yoon BW (2013). Mechanisms of functional recovery after stroke. Front. Neurol. Neurcosci..

[29] Faraji J, Schjetnan AGP, Luczak A, Metz GA (2013). Beyond the Silence: Bilateral somatosensory stimulation enhances skilled movement quality and neural density in intact behaving rats. Behav. Brain. Res..

[30] Fujimoto S, Yamaguchi T, Otaka Y, Kondo K, Tanaka S (2014). Dual-Hemisphere transcranial direct current stimulation improves performance in a tactile spatial discrimination task. Clin. Neurophysiol..

[31] Fujimoto S (2016). Transcranial direct current stimulation over the primary and secondary somatosensory cortices transiently improves tactile spatial discrimination in stroke patients. Front. Neurosci..

[32] Muffel T (2019). Anodal transcranial direct current stimulation over S1 differentially modulates proprioceptive accuracy in young and old adults. Front. Aging Neurosci..

[33] Ragert P, Vandermeeren Y, Camus M, Cohen LG (2008). Improvement of spatial tactile acuity by transcranial direct current stimulation. Clin. Neurophysiol..

[34] Rogalewski A, Breitenstein C, Nitsche MA, Paulus W, Knecht S (2004). Transcranial direct current stimulation disrupts tactile perception. Eur. J. Neurosci..

[35] Sánchez-León CA (2021). Immediate and after effects of transcranial direct-current stimulation in the mouse primary somatosensory cortex. Sci. Rep..

[36] Kunori N, Takashima I (2019). Evaluation of acute anodal direct current stimulation-induced effects on somatosensory-evoked responses in the rat. Brain Res..

[37] Márquez-Ruiz J (2012). Transcranial direct-current stimulation modulates synaptic mechanisms involved in associative learning in behaving rabbits. Proc. Natl. Acad. Sci. USA.

[38] Platz T, Adler-Wiebe M, Roschka S, Lotze M (2018). Enhancement of motor learning by focal intermittent theta burst stimulation (iTBS) of either the primary motor (M1) or somatosensory area (S1) in healthy human subjects. Restor. Neurol. Neurosci..

[39] Ghilardi M (2008). Patterns of regional brain activation associated with different forms of motor learning. Brain Res..

[40] Ghilardi MF, Moisello C, Silvestri G, Ghez C, Krakauer JW (2009). Learning of a sequential motor skill comprises explicit and implicit components that consolidate differently. J Neurophysiol..

[41] Craig JC, Johnson KO (2000). The two-point threshold: not a measure of tactile spatial resolution. Curr. Dir. Psychol. Sci..

[42] Dunn W (2013). Somatosensation assessment using the NIH toolbox. Neurology.

[43] Caparelli-Daquer EM (2012). A Pilot study on effects of 4×1 high-Definition tDCS on motor cortex excitability. Annu. Int. Conf. IEEE Eng. Med. Biol. Soc..

[44] Datta A (2009). precise head model of transcranial direct current stimulation: improved spatial focality using a ring electrode versus conventional rectangular pad. Brain Stimul..

[45] Datta A, Zhou X, Su Y, Parra LC, Bikson M (2013). Validation of finite element model of transcranial electrical stimulation using scalp potentials: implications for clinical dose. J. Neural Eng..

[46] Kuo H (2013). Comparing cortical plasticity induced by conventional and high-definition 4 × 1 ring tDCS: A neurophysiological study. Brain Stimul..

[47] Russell M, Goodman T, Wang Q, Groshong B, Lyeth BG (2014). Gender differences in current received during transcranial electrical stimulation. Front. Psychiatry.

[48] Thomas C, Ghodratitoostani I, Delbem ACB, Ali A, Datta A (2019). Influence of gender-related differences in transcranial direct current stimulation: A computational study. Annu. Int. Conf. IEEE Eng. Med. Biol. Soc..

[49] Schloemer N, Lenz M, Tegenthoff M, Dinse HR, Höffken O (2020). Parallel modulation of intracortical excitability of somatosensory and visual cortex by the gonadal hormones estradiol and progesterone. Sci. Rep..

[50] Adenzato M (2019). Aging, sex and cognitive theory of mind: A transcranial direct current stimulation study. Sci. Rep..

[51] Fehring DJ (2021). Investigating the sex-dependent effects of prefrontal cortex stimulation on response execution and inhibition. Biol. Sex. Differ..

[52] Gorbet DJ, Staines WR (2011). Inhibition of contralateral premotor cortex delays visually guided reaching movements in men but not in women. Exp. Brain Res..

[53] Kuo MF, Paulus W, Nitsche M (2006). Sex differences in cortical neuroplasticity in humans. NeuroReport.

[54] León JJ (2020). Transcranial direct current stimulation improves risky decision making in women but not in men: a sham-controlled study. Behav. Brain Res..

[55] Martin AK, Huang J, Hunold A, Meinzer M (2017). Sex mediates the effects of high-definition transcranial direct current stimulation on "mind-reading". Neuroscience.

[56] Rudroff T, Workman CD, Fietsam AC, Kamholz J (2020). Response variability in transcranial direct current stimulation: Why sex matters. Front. Psychiatry.

[57] Inghilleri M (2004). Ovarian hormones and cortical excitability. An rTMS study in humans. Clin. Neurophysiol..

[58] Smith MJ, Adams LF, Schmidt PJ, Rubinow DR, Wassermann EM (2002). Effects of ovarian hormones on human cortical excitability. Ann. Neurol..

[59] Hanlon CA, McCalley DM (2022). Sex/gender as a factor that influences transcranial magnetic stimulation treatment outcome: Three potential biological explanations. Front. Psychiatry.

[60] Kim S, Stephenson MC, Morris PG, Jackson SR (2014). tDCS-induced alterations in GABA concentration within primary motor cortex predict motor learning and motor memory: A 7 T magnetic resonance spectroscopy study. Neuroimage.

[61] Stagg CJ, Bachtiar V, Johansen-Berg H (2011). The role of GABA in human motor learning. Curr Biol..

[62] Keenan PA, Lindamer LA, Jong SK (1995). Menstrual phase-independent retrieval deficit in women with PMS. Biol. Psychiatry.

[63] Maki PM, Rich JB, Rosenbaum RS (2002). Implicit memory varies across the menstrual cycle: estrogen effects in young women. Neuropsychologia.

[64] Zoghi M, Vaseghi B, Bastani A, Jaberzadeh S, Galea MP (2015). The effects of sex hormonal fluctuations during menstrual cycle on cortical excitability and manual dexterity (a pilot study). PLoS ONE.

[65] Greeley B, Barnhoorn JS, Verwey WB, Seidler RD (2020). Multi-session transcranial direct current stimulation over primary motor cortex facilitates sequence learning, chunking, and one year retention. Front. Hum. Neurosci..

[66] Ljubisavljevic M, Maxood K, Bjekic J, Oommen J, Nagelkerke N (2016). Long-term effects of repeated prefrontal cortex transcranial direct current stimulation (tDCS) on food craving in normal and overweight young adults. Brain Stimul..

[67] Meeker TJ (2019). Non-invasive motor cortex neuromodulation reduces secondary hyperalgesia and enhances activation of the descending pain. Front. Neurosci..

[68] Korman M (2007). Daytime sleep condenses the time course of motor memory consolidation. Nat. Neurosci..

[69] Friedman J, Korman M (2016). Offline optimization of the relative timing of movements in a sequence is blocked by retroactive behavioral interference. Front. Hum. Neurosci..

[70] Yamaguchi T (2020). Transcranial direct-current stimulation combined with attention increases cortical excitability and improves motor learning in healthy volunteers. J. Neuroeng. Rehabil..

[71] Klomjai W, Katz R, Lackmy-valle A (2015). Basic principles of transcranial magnetic stimulation (TMS) and repetitive TMS (RTMS). Ann. Phys. Rehabil. Med..

[72] Shilo G, Lavidor M (2019). Non-linear effects of cathodal transcranial direct current stimulation (tDCS) of the primary motor cortex on implicit motor learning. Exp. Brain Res..

[73] Platz T (2012). Prolonged motor skill learning–a combined behavioural training and θ burst TMS study. Restor. Neurol. Neurosci..

[74] Neva JL (2019). The effects of five sessions of continuous theta burst stimulation over contralesional sensorimotor cortex paired with paretic skilled motor practice in people with chronic stroke. Restor. Neurol. Neurosci..

[75] Wiethoff S, Hamada M, Rothwell JC (2014). Variability in response to transcranial direct current stimulation of the motor cortex. Brain stimul..

[76] Kinnischtzke AK, Simons DJ, Fanselow EE (2014). Motor cortex broadly engages excitatory and inhibitory neurons in somatosensory barrel cortex. Cereb. Cortex.

[77] Kinnischtzke AK, Fanselow EE, Simons DJ (2016). Target-specific M1 inputs to infragranular S1 pyramidal neurons. J. Neurophysiol..

[78] Bradnam LV, Stinear CM, Byblow WD (2011). Cathodal transcranial direct current stimulation suppresses ipsilateral projections to presumed propriospinal neurons of the proximal Upper Limb. J. Neurophysiol..

[79] McCambridge AB, Stinear JW, Byblow WD (2014). A dissociation between propriospinal facilitation and inhibition after bilateral transcranial direct current stimulation. J. Neurophysiol..

[80] Khoshnejad M, Piché M, Saleh S, Duncan G, Rainville P (2014). Serial processing in primary and secondary somatosensory cortex: A DCM analysis of human fMRI data in response to innocuous and noxious electrical stimulation. Neurosci. Lett..

[81] Ploner M, Schoffelen JM, Schnitzler A, Gross J (2009). Functional integration within the human pain system as revealed by Granger causality. Hum. Brain Mapp..

[82] Zhang M (2005). Tactile discrimination of grating orientation: fMRI activation patterns. Hum. Brain Mapp..

[83] Karni A (1998). The acquisition of skilled motor performance: fast and slow experience-driven changes in primary motor cortex. Proc. Natl. Acad. Sci. USA.

[84] Gorbet DJ, Sergio LE (2007). Preliminary sex differences in human cortical BOLD fMRI activity during the preparation of increasingly complex visually guided movements. Eur. J. Neurosci..

[85] Jordan K, Wüstenberg T, Heinze HJ, Peters M, Jäncke L (2002). Women and men exhibit different cortical activation patterns during mental rotation tasks. Neuropsychologia.

[86] Krause B, Cohen Kadosh R (2014). Not all brains are created equal: the relevance of individual differences in responsiveness to transcranial electrical stimulation. Front. Syst. Neurosci..

[87] Bhattacharjee S (2021). Sex difference in tDCS current mediated by changes in cortical anatomy: a study across young, middle and older adults. Brain stimul..

[88] Russell MJ (2017). Sex and electrode configuration in transcranial electrical stimulation. Front. Psychiatry.

[89] Amunts K, Jancke L, Mohlberg H, Zilles K (2000). Interhemispheric asymmetry of the human motor cortex related to handedness and gender. Neuropsychologia.

[90] Gennatas ED (2017). Age-related effects and sex differences in gray matter density, volume, mass, and cortical thickness from childhood to young adulthood. J. Neurosci..

[91] Ruigrok AN (2014). A meta-analysis of sex differences in human brain structure. Neurosci. Biobehav. Rev..

[92] Li LM, Uehara K, Hanakawa T (2015). The contribution of interindividual factors to variability of response in transcranial direct current stimulation studies. Front. Cell. Neurosci..

[93] Dorfberger S, Japha EA, Karni A (2009). Sex differences in motor performance and motor learning in children and adolescents: An Increasing male advantage in motor learning and consolidation phase gains. Behav. Brain Res..

[94] Ingram LA (2019). The upper limb physiological profile assessment: description, reliability, normative values and criterion validity. PLoS ONE.

[95] Oxford Grice K (2003). Adult norms for a commercially available nine hole peg test for finger dexterity. Am. J. Occup. Ther..

[96] Piek JP, Gasson N, Barrett N, Case I (2002). Limb and gender differences in the development of coordination in early infancy. Hum. Mov. Sci..

[97] Thomas JR, French KE (1985). Gender differences across age in motor performance a meta-analysis. Psychol. Bull..

[98] Ngun TC, Ghahramani N, Sánchez FJ, Bocklandt S, Vilain E (2011). The genetics of sex differences in brain and behavior. Front. Neuroendocrinol..

[99] Dewing P (2006). Direct regulation of adult brain function by the male-specific factor SRY. Curr. Biol..

[100] Laakso I, Tanaka S, Koyama S, Saints VD, Hirata A (2015). Inter-subject variability in electric fields of motor cortical tDCS. Brain Stimul..

[101] Khedr EM, Abdelrahman AA, Safwat SM, Moheb A, Noaman MM (2021). The effect of acute and chronic nicotine consumption on intra-cortical inhibition and facilitation: A transcranial magnetic stimulation study. Neurophysiol Clin..

[102] Thirugnanasambandam N (2011). Nicotinergic impact on focal and non-focal neuroplasticity induced by non-invasive brain stimulation in non-smoking humans. Neuropsychopharmacology.

[103] Clayton JA, Tannenbaum C (2016). Reporting sex, gender, or both in clinical research?. JAMA.

