# Supplementary material for: Sensorimotor performance after high-definition transcranial direct current stimulation over the primary somatosensory or motor cortices in men versus women
Source: Sci Rep. 2022 Jul 1;12:11117. doi: 10.1038/s41598-022-15226-2 (PMC9249866; doi:10.1038/s41598-022-15226-2)
Supplement: Supplementary file 1 — Supplementary Table 1. [file 41598_2022_15226_MOESM1_ESM.docx]

Supplementary Table 1:

Results of mixed design ANOVA with time (pretest, posttest) as the within-subject factor, group (S1, M1, sham) and sex (male, female) as the between subject-factors for the motor and sensation tasks

| **Motor task** | | | | | | | | | | | | |
| --- | --- | --- | --- | --- | --- | --- | --- | --- | --- | --- | --- | --- |
| Variable | Main effects and interactions | | | | | | | Relevant post hoc comparisons | | | | |
|  | Time | Group | Sex | Time X Group | Time X Sex | Sex X Group | Sex X Group X Time | Time | Sex | Time X Group | Time X Sex | Sex X Group X Time |
| Reaction time (s) | F(1,39) = 3.283; p = 0.078; partial η2 = 0.08; observed power = 0.42 | F(2,39) = 0.575; p = 0.567; partial η2 = 0.03; observed power = 0.14 | F(1,39) = 5.206; **p = 0.028**; partial η2 = 0.12; observed power = 0.61 | F(2,39) = 0.208; p = 0.813; partial η2 = 0.01; observed power = 0.08 | F(1,39) = 0.341; p = 0.563; partial η2 = 0.01; observed power = 0.09 | F(2,39) = 0.238; p = 0.789; partial η2 = 0.01; observed power = 0.09 | F(2,39) = 4.419; **p = 0.019**; partial η2 = 0.19; observed power = 0.73 | NR | Women (faster) vs. men, **p = 0.028** | NR | NR | In men,  Group x Time (F(2,18) = 3.914; **p = 0.039**; partial η2 = 0.30; observed power = 0.63) (only in M1, reaction time decreased significantly in posttest vs. pretest, p = 0.006);  In women,  Time (F(2,21) = 2.205; p = 0.135; partial η2 = 0.17; observed power = 0.40) |
| Movement time (s) | F(1,39) = 13.550; **p = 0.001**; partial η2 = 0.26; observed power = 0.95 | F(2,39) = 0.232; p = 0.794; partial η2 = 0.01; observed power = 0.08 | F(1,39) = 2.779; p = 0.102; partial η2 = 0.07; observed power = 0.37 | F(2,39) = 1.119; p = 0.337; partial η2 = 0.05; observed power = 0.23 | F(1,39) = 12.460, **p = 0.001**; partial η2 = 0.24; observed power = 0.93 | F(2,39) = 0.250; p = 0.780; partial η2 = 0.01; observed power = 0.09 | F(2,39) = 0.609; p = 0.549; partial η2 = 0.03; observed power = 0.14 | Posttest (decreased) vs. pretest, **p = 0.001** | NR | NR | In men,  Posttest vs. pretest, p = 0.903;  In Women,  Posttest (decreased) vs. pretest, **p < 0.001** | NR |
| Endpoint error (cm) | F(1,39) = 4.483; **p = 0.041**; partial η2= 0.10; observed power = 0.54 | F(2,39) = 0.677; p = 0.514; partial η2 = 0.03; observed power = 0.16 | F(1,39) = 14.005; **p = 0.001**; partial η2 = 0.26; observed power = 0.95 | F(2,39) = 0.361; p = 0.699; partial η2= 0.02; observed power = 0.10 | F(1,39) = 6.335; **p= 0.016**; partial η2 = 0.14; observed power = 0.69 | F(2,39) = 0.21; p = 0.808; partial η2 = 0.01; observed power = 0.08 | F(2,39) = 1.155; p = 0.325; partial η2= 0.06; observed power = 0.24 | Posttest (smaller) vs. pretest, **p = 0.001** | Women (smaller) vs. men, **p = 0.001** | NR | In men, Posttest (smaller) vs. pretest, **p = 0.006**;  In women,  Posttest vs. pretest. p = 0.772 | NR |
| **Sensation task - TPOD** | | | | | | | | | | | | |
| Variable | Main effects and interactions | | | | | | | Relevant post hoc comparisons | | | | |
|  | Time | Group | Sex | Time X Group | Time X Sex | Sex X Group | Sex X Group X Time | Time | Sex | Time X Group | Time X Sex | Sex X Group X Time |
| Percent of correct trials | F(1,39) = 8.149; **p = 0.007**; partial η2 = 0.17; observed power =0.80 | F(2,39) = 2.984; p= 0.062; partial η2 = 0.13; observed power = 0.55 | F(1,39) = 0.069; p= 0.795; partial η2 = 0.00; observed power = 0.06 | F(2,39) = 2.765; p= 0.075; partial η2 = 0.12; observed power = 0.51 | F(1,39) = 1.285; p= 0.264; partial η2 = 0.03; observed power = 0.20 | F(2,39) = 0.598; p= 0.555; partial η2 = 0.03; observed power = 0.14 | F(2,39) = 1.272; p= 0.292; partial η2 = 0.06; observed power = 0.26 | Posttest (higher) vs. pretest, **p = 0.007** | NR | In S1,  Posttest (higher) vs. pretest, **p = 0.012**;  In M1,  Posttest vs. pretest; p = 0.613; In sham; Posttest vs. pretest, p= 0.294.  In posttest,  S1 (higher) vs. M1, **pBonferroni= 0.009**;  S1 vs. sham, pBonferroni= 0.079;  M1 vs. sham, pBonferroni= 1.000 | NR | NR |
| **Sensation task - Proprioception** | | | | | | | | | | | | |
| Variable | Main effects and interactions | | | | | | | Relevant post hoc comparisons | | | | |
|  | Time | Group | Sex | Time X Group | Time X Sex | Sex X Group | Sex X Group X Time | Time | Sex | Time X Group | Time X Sex | Sex X Group X Time |
| Movement time (s) | F(1,39) = 26.688; **p < 0.001**; partial η2 = 0.41; observed power = 0.99 | F(2,39) = 0.077; p = 0.926; partial η2 = 0.00; observed power = 0.06 | F(1,39) = 0.192; p = 0.664; partial η2 = 0.01; observed power = 0.07 | F(2,39) = 1.141; p = 0.869; partial η2 = 0.01; observed power = 0.07 | F(1,39) = 3.609; p = 0.065; partial η2 = 0.09; observed power = 0.46 | F(2,39) = 0.977; p = 0.386; partial η2 = 0.05; observed power = 0.21 | F(2,39) = 0.433; p = 0.652; partial η2 = 0.02; observed power = 0.12 | Posttest (shorter) vs. pretest, **p < 0.001** | NR | NR | NR | NR |
| Endpoint error (s) | F(1,39) = 0.201; p = 0.657; partial η2 = 0.01; observed power = 0.07 | F(2,39) = 0.329; p = 0.721; partial η2 = 0.02; observed power = 0.10 | F(1,39) = 0.908; p = 0.346; partial η2 = 0.02; observed power = 0.16 | F(2,39) = 0.367; p = 0.695; partial η2 = 0.02; observed power = 0.11 | F(1,39) = 0.773; p = 0.385; partial η2 = 0.02; observed power = 0.14 | F(2,39) = 3.104; p = 0.056; partial η2 = 0.14; observed power = 0.56 | F(2,39) = 0.696; p = 0.505; partial η2 = 0.03; observed power = 0.16 | NR | NR | NR | NR | NR |

Legend: Post hoc comparisons are mentioned only for significant main and interaction effects. TPOD = Two-point orientation discrimination; NR – not relevant
