# Supplementary material for: Sensorimotor performance after high-definition transcranial direct current stimulation over the primary somatosensory or motor cortices in men versus women
Source: Sci Rep. 2022 Jul 1;12:11117. doi: 10.1038/s41598-022-15226-2 (PMC9249866; doi:10.1038/s41598-022-15226-2)
Supplement: Supplementary file 2 — Supplementary Table 2. [file 41598_2022_15226_MOESM2_ESM.docx]

Supplementary Table 2:

Results of ANCOVA with time (pretest, posttest) as the within-subject factor, group (S1, M1, sham) as the between subject-factor and sex (male, female) as the covariate factor for the motor and sensation tasks

| **Motor task** | | | | | | | | | |
| --- | --- | --- | --- | --- | --- | --- | --- | --- | --- |
| Variable | Main effects and interactions | | | | | Relevant post hoc comparisons | | | |
|  | Time | Sex | Group | Time X Group | Time X Sex | Time | Sex | Group | Time X Sex |
| Reaction time (s) | F(1,41) = 1.287; p = 0.263; partial η2 = 0.03; observed power = 0.20 | F(1,41) = 5.418; **p = 0.025**; partial η2 = 0.12; observed power = 0.62 | F(2,41) = 0.605; p = 0.551; partial η2 = 0.03; observed power = 0.14 | F(2,41) = 0.402; p = 0.672; partial η2 = 0.02; observed power = 0.11 | F(1,41) = 0.343; p = 0.561; partial η2 = 0.01; observed power = 0.09 | NR | Women (faster) vs. men, p = 0.025 | NR | NR |
| Movement time (s) | F(1,41) = 4.460; **p = 0.041**; partial η2 = 0.10; observed power = 0.54 | F(1,41) = 2.883; p = 0.097; partial η2 = 0.07; observed power = 0.32 | F(2,41) = 0.231; p = 0.795; partial η2 = 0.01; observed power = 0.08 | F(2,41) = 0.973; p = 0.387; partial η2 = 0.05; observed power = 0.21 | F(1,41) = 12.570, **p = 0.001**; partial η2 = 0.24; observed power = 0.93 | Posttest (decreased) vs. pretest, **p < 0.001** | NR | NR | In men,  Posttest vs. pretest, p = 0.903  In Women,  Posttest (decreased) vs. pretest, **p < 0.001** |
| Endpoint error (cm) | F(1,41) = 9.332; **p = 0.004**; partial η2= 0.19; observed power = 0.85 | F(1,41) = 14.556; **p = 0.000**; partial η2 = 0.26; observed power = 0.96 | F(2,41) = 0.704; p = 0.500; partial η2 = 0.03; observed power = 0.16 | F(2,41) = 0.302; p = 0.741; partial η2= 0.02; observed power = 0.10 | F(1,41) = 6.419; **p= 0.015**; partial η2 = 0.14; observed power = 0.70 | Posttest (smaller) vs. pretest, **p = 0.043** | Women (smaller) vs. men, **p = 0.000** | NR | In men, Posttest (smaller) vs. pretest, **p = 0.006**;  In women,  Posttest vs. pretest. p = 0.772 |
| **Sensation task - TPOD** | | | | | | | | | |
| Variable | Main effects and interactions | | | | | Relevant post hoc comparisons | | | |
|  | Time | Sex | Group | Time X Group | Time X Sex | Time | Sex | Group | Time X Sex |
| Percent of correct trials | F(1,41) = 3.609; p = 0.064; partial η2 = 0.08; observed power =0.46 | F(1,41) = 0.080; p = 0.779; partial η2 = 0.002; observed power =0.06 | F(2,41) = 3.392; **p = 0.043**; partial η2 = 0.142; observed power = 0.61 | *F(2,41) = 2.367; p = 0.106; partial η2 = 0.10; observed power = 0.45 | (1,41) = 1.228; p = 0.274; partial η2 = 0.03; observed power = 0.19 | NR | NR | S1 (higher) vs. M1, **pBonferroni = 0.044** | NR |
| **Sensation task - Proprioception** | | | | | | | | | |
| Variable | Main effects and interactions | | | | | Relevant post hoc comparisons | | | |
|  | Time | Sex | Group | Time X Group | Time X Sex | Time | Sex | Group | Time X Sex |
| Movement time (s) | F(1,41) = 11.986**; p = 0.001**; partial η2 = 0.23; observed power = 0.92 | F(1,41) = 0.171; p = 0.681; partial η2 = 0.00; observed power = 0.07 | F(2,41) = 0.028; p = 0.973; partial η2 = 0.00; observed power = 0.05 | F(2,41) = 1.192; p = 0.826; partial η2 = 0.01; observed power = 0.08 | F(1,41) = 3.760; p = 0.059; partial η2 = 0.08; observed power = 0.47 | Posttest (shorter) vs. pretest, **p = 0.001** | NR | NR | NR |
| Endpoint error (s) | F(1,41) = 0.873; p = 0.356; partial η2 = 0.02; observed power = 0.15 | F(1,41) = 0.861; p = 0.359; partial η2 = 0.02; observed power = 0.15 | F(2,41) = 0.441; p = 0.647; partial η2 = 0.02; observed power = 0.12 | F(2,41) = 0.379; p = 0.687; partial η2 = 0.02; observed power = 0.11 | F(1,41) = 0.777; p = 0.383; partial η2 = 0.02; observed power = 0.14 | NR | NR | NR | NR |

Legend: Post hoc comparisons are mentioned only for significant main and interaction effects. TPOD = Two-point orientation discrimination; NR – not relevant; *For comparison with the results of mixed design ANOVA (described in the Results section and Supplementary Table 1), despite the non-significant effect of Time X Group for TPOD, separate analyses were conducted for each group and showed the following results: 1. Only in S1, the proportion of correct trials tended to increase in posttest compared to pretest p = 0.068; In M1 p = 0.221; In sham p= 0.730; 2. In posttest, the proportion of correct trials increased only in S1 vs. M1 pBonferroni= 0.009; the proportion of correct trials tended to increase in S1 vs. sham, pBonferroni= 0.079; M1 vs. sham, pBonferroni= 1.000
